# Association of geriatric nutritional risk index with the risk of osteoporosis in the elderly population in the NHANES

**DOI:** 10.3389/fendo.2022.965487

**Published:** 2022-11-29

**Authors:** Wei Huang, Yingqi Xiao, Hongwei Wang, Kaixiang Li

**Affiliations:** ^1^ Department of Orthopaedics, Dongguan Tungwah Hospital, Dongguan, China; ^2^ Department of Pulmonary and Critical Care Medicine, Dongguan Tungwah Hospital, Dongguan, China

**Keywords:** geriatric nutritional risk index, osteoporosis, risk factors, older adults, NHANES

## Abstract

**Background:**

Osteoporosis is common in the elderly, and malnutrition is considered a major risk factor for osteoporosis. This study investigated the relationship between the Geriatric Nutrition Risk Index (GNRI) and osteoporosis based on a large cross-sectional study of the National Health and Nutrition Examination Survey (NHANES).

**Methods:**

We included 7405 older adults from NHANES (2005 to 2018) and divided them into the High-GNRI and Low-GNRI groups based on GNRI levels to compare the prevalence of osteoporosis among the two groups. A multi-factor logistic regression analysis was used to determine whether GNRI was an independent risk factor for osteoporosis. Spearman’s rank correlation coefficient was computed to investigate the linear relationship between geriatric nutritional risk index (GNRI) and bone mineral density (BMD) T-score. Finally, a generalized additive model (GAM) revealed whether there was a non-linear relationship between GNRI and osteoporosis.

**Results:**

The prevalence of osteoporosis was higher in the Low-GNRI group than those in the High-GNRI group (12.2% vs. 8.2%; *P* = 0.001). Similarly, the femoral neck BMD T-scores (-1.09 ± 1.42 vs. -0.91 ± 1.31; *P* = 0.003). However, there was no significant difference between Low-GNRI group and High-GNRI group in lumbar BMD T-scores (1.700 ± 1.69 vs 1.85 ± 1.72; *P*>0.05). The multi-factor logistic regression analysis identified low GNRI as an independent risk factor for osteoporosis (OR: 1.544; 95% CI: 1.179-2.022; *P* < 0.001). Besides, GNRI showed a positive linear correlation (*P* < 0.001) with femoral neck BMD T-scores in older adults, with a progressive trend towards higher BMD as GNRI increased. By contrast, there was no linear correlation between GNRI and lumbar BMD T-score (*P* = 0.978). Lastly, the dose response curve revealed the non-linear negative correlation between GNRI and the risk of osteoporosis in the elderly (non-linear P < 0.001). With the increase of GNRI, the risk of osteoporosis gradually decreased, especially when GNRI was greater than 100, the downward trend was more significant.

**Conclusion:**

GNRI is an independent risk factor for osteoporosis in the elderly and is negatively and non-linearly associated with the risk of osteoporosis in the elderly population.

## Introduction

Osteoporosis causes patients’ bone mineral density (BMD) and bone quality to decrease, resulting in a variety of fractures throughout the body (1, 2). In the elderly, osteoporotic fractures affect up to 50% of women and 20% of men over the age of 50 (3). Therefore, identifying risk factors associated with osteoporosis is essential for its prevention and treatment.

Geriatric nutritional risk index (GNRI), first reported in 2005 (4), is a simple dietary index strongly associated with the prognosis of many diseases such as diabetes, heart failure, and cancer (5–7). Various studies (8) have found a significant correlation between GNRI and bone mineral density and osteoporosis. In the Chinese population, the GNRI value was increased with the BMD level (8). Similarly, GNRI was positively correlated with BMD and negatively correlated with the incidence of osteoporosis in T2 diabetic patients (5). In hemodialysis patients, GNRI was significantly associated with BMD of the femoral neck, lumbar spine, and distal radius, and combining GNRI with traditional risk factors (age, sex, diabetes, and cardiovascular disease) accurately predicted patient mortality (9). Moreover, a low GNRI level is also considered a risk factor for decreased bone mineral density in the femoral neck in young men with rheumatoid arthritis (10). The studies mentioned above confirm that GNRI is strongly linked to osteoporosis and bone mineral density. Nonetheless, the studies generally include small populations (fewer than a few hundred people), are primarily single-center studies, and focus on older men.

The National Health and Nutrition Examination Survey (NHANES) is a large-scale population-based cross-sectional survey that collects information about the health and nutrition of the U.S. household population. NHANES database has broad sample coverage and various indicators that provide access to demographics, socioeconomics, diet and health, physiological measurements, laboratory tests, and other information throughout the US. In the present study, we identify the correlation between GNRI and the risk of osteoporosis in the elderly population with the help of the NHANES large-scale cross-sectional study, which can be used as a reference for the prevention of osteoporosis.

## Methods

### Database and survey populations

The data used in this study were acquired from NHANES (https://www.cdc.gov/nchs/nhanes/index.htm) website. This is a cross-sectional survey conducted by the National Center for Health Statistics (NCHS) and the Centers for Disease Control and Prevention. NHANES is designed to provide nationally representative data on the civilian population of the U.S. The NCHS Ethics Review Board approved the data collection protocol, and all survey participants gave informed consent before being interviewed and examined. For this study, a dataset was constructed using publicly available data files of NHANES responses from 2005 to 2018. The study population included all NHANES respondents.

### GNRI evaluation and grouping

According to previous research, the GNRI was calculated using the subject’s height (cm), weight (kg), ideal weight (kg), and serum albumin (g/L) (4, 5). Calculation formula: GNRI = (1.489) × Albumin (g/L) + 41.7 × [body weight/ideal body weight], ideal body mass = 22 × Height (m) × Height (m). When the weight exceeds the ideal weight, set the weight/ideal weight = 1. GNRI nutritional assessment level determination: High nutritional risk (GNRI < 98), low nutritional risk (GNRI ≥ 98) (4, 7). The patients included in this study were divided into two groups: High-GNRI group (GNRI ≥ 98) and Low-GNRI group (GNRI < 98).

### Osteoporosis assessment

The World Health Organization (WHO) (11), defines osteoporosis as failure to meet one of the following conditions: (1) previous self-reported history of osteoporosis as determined by a physician’s diagnosis; (2) no self-reported history of osteoporosis, but laboratory dual-energy x-ray absorptiometry (DXA) showing a femoral neck or lumbar spine (L1-3) T-score ≤ -2.5 (12, 13).

### Demographic characteristics

Demographic characteristics such as age, gender, and race were confounding factors between exposure and the primary outcome. The socioeconomic covariates comprise education level, marital status, and health insurance coverage. Data on health-related behaviors, such as smoking, alcohol consumption, physical activity, and history of glucocorticoid use, were also collected. Physical activity during leisure time for the past 30 days was assessed based on questionnaire data to determine their level of physical activity, frequency, and duration of each exercise session. Metabolic equivalent (MET) scores were calculated for the average physical activity level over the past 30 days based on the recommended MET scores provided for each response in the questionnaire section of the NHANES methodology. Similarly, medical comorbidity variables were also acquired, including body mass index (BMI), blood calcium, glomerular filtration rate (GFR), hypertension, diabetes, and cancer. For the missing covariate data, we used the variable missing interpolation method to supplement the missing data through the R software MI program.

### Statistical analysis

Data were analyzed using χ^2^ test to analyze weighted differences in cohort characteristics and outcome variables between exposure groups. For preliminary analysis, multifactorial logistic regression was used to determine the association between exposure and outcome variables. All descriptive studies were tested for significance using two-sided tests at a significance level of *P* < 0.05. Finally, a generalized additive model (GAM) was used to examine the nonlinear relationship between the outcome variable and the exposure factors. Moreover, Spearman correlation analysis was performed to investigate the correlation between GNRI and BMD. All data analyses were computed using Empower Stats software (www.empowerstats.com, X&Y solutions, Inc. Boston MA) and R.3.5.2 (http://www.R-project.org). Furthermore, sample sizes were based on available data, and no ex-ante minimum sample size calculations were performed.

## Results

**Figure 1:**
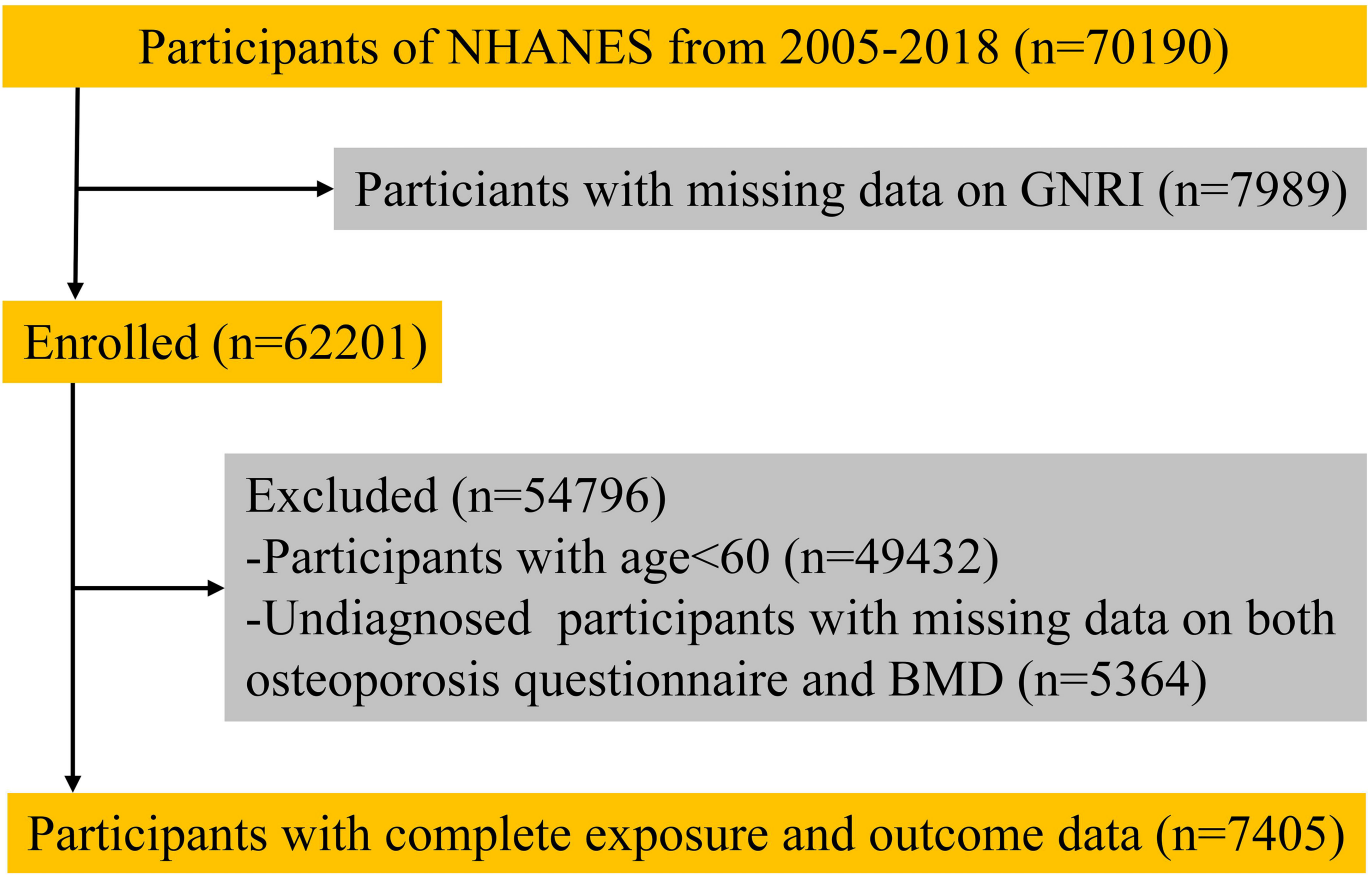
Flow diagram of the screening and selection process.

We collected survey data from 70190 participants. After excluding those who lacked outcome or exposure data, 7450 participants (including 632 with osteoporosis and 6773 in the non-osteoporotic control population) were included in the analysis (**Figure 1**).

### Characteristics of participants in the High-GNRI and Low-GNRI group

The distribution of cohort characteristics stratified by GNRI level degree is shown in **Table 1**. In the preliminary analysis, 12.2% (67/550) of participants in the Low-GNRI group had comorbid osteoporosis compared to 8.2% (565/6855) of participants in the High-GNRI group, with a significant difference between the two groups (*P* = 0.001). The Low-GNRI group had a higher proportion of males, 59.5% (327/550), and significantly higher mean age (71.2 ± 7.3 vs. 69.9 ± 7.1; *P* < 0.001) than the High-GNRI group. Besides, the Low-GNRI group also had a lower BMI (28.6 ± 5.3 vs. 25.4 ± 6.0; *P* < 0.001) than the High-GNRI group. In terms of social factors, the Low-GNRI group had a higher proportion of divorced, separated, or widowed people (44.2% vs 35.0%) than the High-GNRI group. However, the Low-GNRI group had a lower proportion of people with a high school education than the High-GNRI group (61.3% vs 70.6; *P<0.001*). Furthermore, there was also a statistical difference between the two groups regarding hypertension, diabetes mellitus, and cancer history (*P* < 0.05). HbA1c (6.05 ± 1.09 vs. 6.01 ± 1.10; *P* < 0.001) was higher in the High-GNRI group than in the Low-GNRI group, which could be attributed to the higher prevalence of diabetes (31.6% vs. 26.2%; *P* < 0.001) in the High-GNRI group than in the Low-GNRI group as well. Subsequent analysis after direct deletion of all missing data showed that the estimates of the effect of direct deletion of missing values and multiple interpolation were similar.

**Table 1:** Characteristics of participants included in study from the NHANES (2005 to 2018).

Characteristic	High-GNRI (N=6855)	Low-GNRI (N=550)	*P*-value
Age (y)	69.9±7.1	71.2±7.3	<0.001
Male sex	3518 (51.3)	327 (59.5)	<0.001
Race			<0.001
Hispanic	1468 (21.4)	89 (16.2)	
Non-Hispanic white	3636 (53.0)	217 (39. 5)	
Non-Hispanic black	1259 (18.4)	195 (35.5)	
Other	492 (7.2)	49 (8.9)	
Education beyond high school	4839 (70.6)	337 (61.3)	<0.001
Marital status			<0.001
Never married	308 (4.5)	28 (5.1)	
Married or living with partner	4148 (60.5)	279 (50.8)	
Divorced, separated, or widowed	2399 (35.0)	243 (44.2)	
Health insurance coverage	6314 (92.1)	502 (91.3)	0.486
BMI (kg/m2)	28.6±5.3	25.4±6.0	<0.001
eGFR, ml/min per 1.73 m2	74.0±18.9	68.6±20.5	<0.001
ALT (U/L)	22.3±15.8	17.41±16.68	<0.001
AST (U/L)	24.9±11.5	27.9±13.95	<0.001
HbA1c	6.05±1.09	6.01±1.10	0.401
Alcohol user	5664 (82.6)	470 (85.5)	0.091
Smoker	3509 (51.2)	325 (59.1)	<0.001
Glucocorticoid user	424 (6.2)	57 (10.4)	<0.001
Hypertension	4815 (70.2)	389 (70.7)	0.810
Cancer	1351 (19.7)	157 (28.6)	<0.001
Osteoporosis	565 (8.2)	67 (12.2)	0.001
Diabetes	2163 (31.6)	144 (26.2)	0.009
Blood calcium	2.36±0.10	2.32±0.11	<0.001
MET	5280.1±92.5	3738.98±78.3	<0.001

All values are displayed as n (%). χ^2^ analysis is used to test significance between groups for categorical variables. BMI, body mass index; eGFR, estimated glomerular filtration rate; HbA1c, glycosylated Hemoglobin, type A1C; ALT, alanine aminotransferase; AST, aspartate aminotransferase. MET, Metabolic equivalent.

### BMD T-scores distribution of participants in the High-GNRI and Low-GNRI group

**Figure 2:**
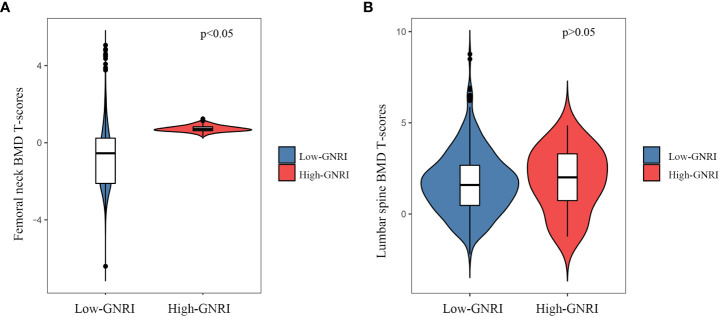
The violin plot of BMD T-scores distribution in High-GNRI and Low-GNRI group subjects. **(A)** Distribution of femoral neck BMD T-scores in two groups. **(B)** Distribution of lumbar BMD T-scores in two groups.

The femoral neck BMD T-scores (-1.09 ± 1.42 vs. -0.91 ± 1.31; *P = 0.003*) in the Low-GNRI group were lower than those in the High-GNRI group (**Figure 2A**). Similarly, the lumbar spine BMD T-scores (1.700± 1.69 vs. 1.85 ± 1.72; *P>0.05*) in the Low-GNRI group were also lower than in the High-GNRI group, but the difference is not significant (**Figure 2B**). Thus, the trend toward lower femoral neck BMD T-scores was temporarily outweighed by the lower GNRI levels.

### GNRI level and osteoporosis

Take High-GNRI as control group, a multifactorial logistic regression analysis was used to determine whether Low-GNRI was a risk factor for osteoporosis. In an unadjusted survey-weighted analysis, the OR for predicting osteoporosis risk by Low-GNRI was 1.544 (*P* = 0.001, **Table 2**). After adjusting for covariates such as age, sex, race, education level, marital status, BMI, smoker, alcohol user, glucocorticoid user, physical activity, blood calcium, hypertension, cancer, diabetes, the OR for predicting osteoporosis risk by Low-GNRI was 1.834 (*P* < 0.001, **Table 2**). In short, it is suggested that Low-GNRI is a risk factor for the development of osteoporosis.

**Table 2:** Logistic regression analysis for associations between Low-GNRI and osteoporosis.

	OR (95% CI)	*P*-value
Un- adjusted	1.544 (1.179-2.022)	0.001
Model 1	1.820 (1.359-2.438)	<0.001
Model 2	1.869 (1.394-2.507)	<0.001
Model 3	1.834 (1.365-2.465)	<0.001

Model 1 was adjusted for age, sex, race.

Model 2 was adjusted for age, sex, race, education level, marital status, BMI, smoker, alcohol user, glucocorticoid user, physical activity.

Model 3 was adjusted for age, sex, race, education level, marital status, BMI, smoker, alcohol user, glucocorticoid user, physical activity, blood calcium, hypertension, cancer, diabetes.

High GNRI was the control group.

Furthermore, subgroup analysis by age, gender, ethnicity, showed consistent results across categorized subgroups of the population, with low levels of GNRI consistently associated with an increased risk of osteoporosis prevalence in the elderly population, all at *P* < 0.05 (**Table 3**).

**Table 3:** Subgroup logistic regression analysis for the association between Low-GNRI and osteoporosis.

	OR (95%CI)	*P*-value	Interaction *P*-value
Age(y)			0.181
60 ~ 69	1.867 (1.500-2.478)	<0.001	
70 ~79	1.420 (1.337-2.367)	<0.001	
≥80	1.886 (1.606-2.910)	<0.001	
Gender			0.920
Female	1.614 (1.146-2.270)	0.006	
Male	2.173 (1.356-3.482)	0.001	
Race			0.317
Hispanic	1.657 (1.360- 2595)	<0.001	
Non-Hispanic white	1.841 (1.785-2281)	<0.001	
Non-Hispanic black	1.229 (1.141-2.562)	<0.001	
Other	1.913 (1.713-3.087)	0.003	

High-GNRI was the control group.

### Linear correlation analysis of BMD T-scores and GNRI in older adults

**Figure 3:**
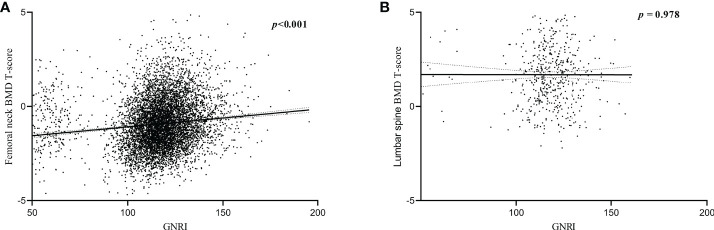
Correlation between BMD T-scores and GNRI. **(A)** Correlation between femoral neck BMD T-scores and GNRI. **(B)** Correlation between lumbar spine BMD T-scores and GNRI.

Results indicated a positive linear correlation between GNRI and BMD T-scores of the femoral neck in older adults (*P* < 0.001), with a gradual increase in BMD T-scores as GNRI increased, but the correlation between lumbar spine BMD T-scores and GNRI was not strong (*P =* 0.978, **Figures 3A, B**).

### Dose-response relationship between GNRI and risk of osteoporosis in older adults

**Figure 4:**
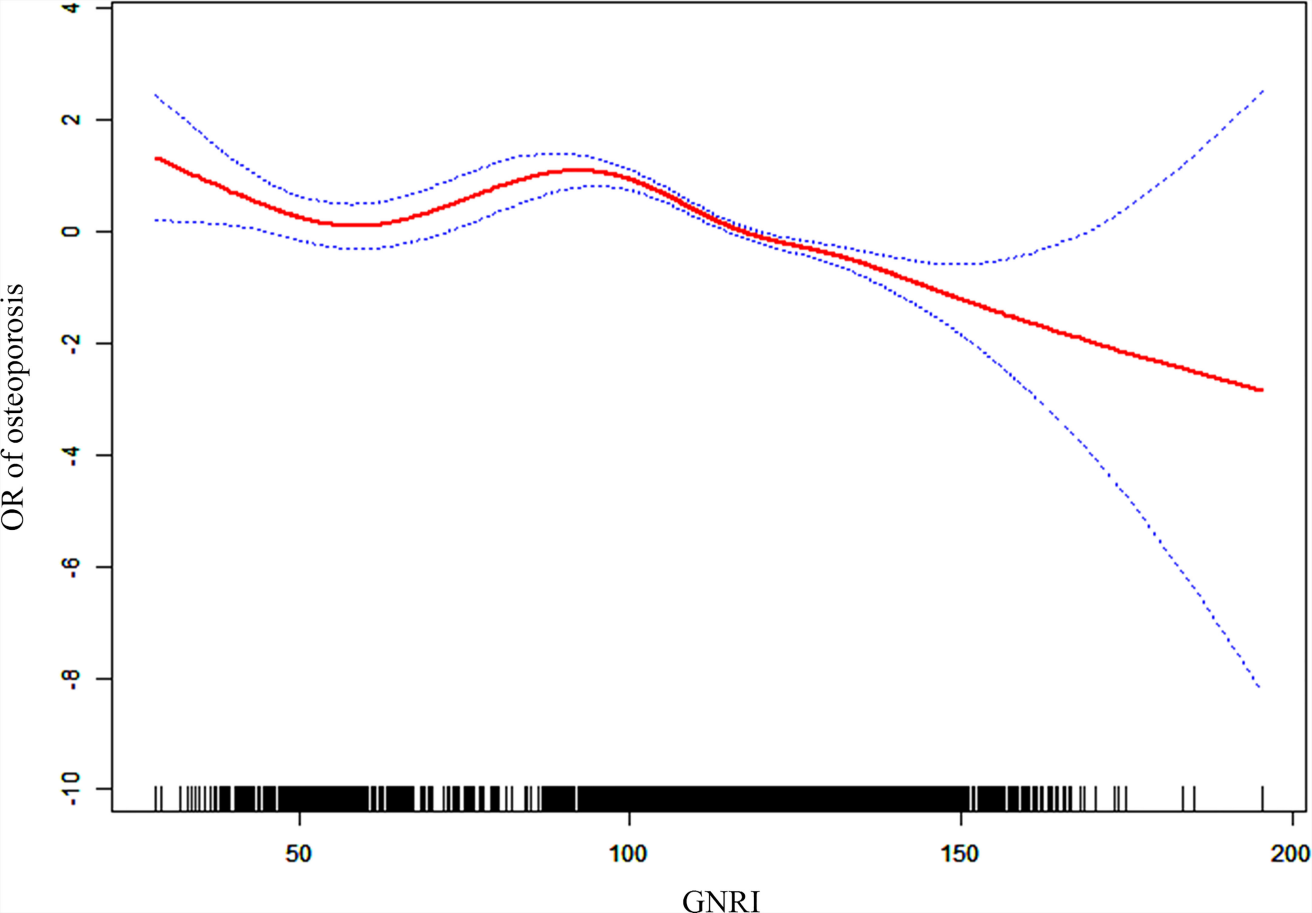
Dose-response association between OR of osteoporosis and GNRI (adjusted for age, sex, race, smoker, alcohol user, glucocorticoid user, hypertension, cancer, and diabetes).

The dose-response curves between GNRI and the risk of osteoporosis revealed a non-linear negative association between GNRI and the risk of osteoporosis in the elderly population (non-linear *P* < 0.001). Similarly, after adjusting covariates (age, sex, ethnicity, and race), a negative association between GNRI and risk of osteoporosis was also found (non-linear *P* < 0.001; **Figure 4**). With the increase of GNRI, the risk of osteoporosis gradually decreased, especially when GNRI was greater than 100, the downward trend was more significant.

## Discussion

Osteoporosis is a complex biological process that primarily involves loss of bone mass and loss of bone strength with increasing age (12). Osteoporosis is characterized by the deterioration of bone microstructure, resulting in increased bone fragility and fracture (1). The occurrence of osteoporosis is closely related to age, gender, height, weight, smoking, alcohol, diabetes, dementia, cancer, asthma or chronic obstructive pulmonary disease, cardiovascular disease, chronic liver disease, and many other physiological or pathological conditions (12).

Chronic alcohol abuse is known to be associated with osteoporosis and the development of osteoporotic fractures. Ethanol promotes elevated P21 expression, while high P21 expression inhibits osteoblast differentiation and mineralization, which further interferes with bone remodeling (14, 15).

Besides, several clinical studies have found a positive association between alcohol consumption and the development of osteoporosis (OR = 2.95, *P* < 0.05). Compared to abstainers, those who drank 0.5-1 drink per day had 1.38 times the risk of developing osteoporosis (OR = 1.38, *P* < 0.05) and 1-2 drinks per day (OR = 1.34, *P* < 0.05); those who drank two or more drinks per day had 1.63 times the risk of developing osteoporosis (OR = 1.63, *P* < 0.05) (16). In addition, smoking research can also lead to osteoporosis, and a study by the Taiwan Biobank found that the smoking-only group was more likely to develop osteoporosis than non-smoking participants (OR=1.24, P=0.003) (17). Smoking has an effect on bone integrity, where this adverse effect is mainly attributed to nicotine, one of the main components of the particulate phase of tobacco smoke (18). Smoking alters bone remodeling, including altering osteoblast bone formation, increasing osteoclast osteoblastic degeneration, or both. Clinical studies have also found that smoking significantly reduces bone density in the femoral neck and lumbar spine, and epidemiological studies have shown that smokers lose more cortical bone than nonsmokers (19).

Our study also observed that the Low-GNRI group had a higher prevalence of smoking (59.1% vs. 51.2%; P < 0.001) and alcohol consumption (85.5% vs. 82.6%; P = 0.091) compared to the High-GNRI group. Therefore, in a subsequent logistic regression, we adjusted for smoking as well as alcohol as covariates, and the results still supported a significantly higher risk of osteoporosis in the Low-GNRI population than in the High-GNRI.

Adequate nutrition plays an essential role in the stability of bone structure. Malnutrition often leads to lower daily activities and longer hospital stays and recovery times for the elderly (20). Studies suggested that high protein intake positively affects bone mineral density or content (21). In contrast, lower levels of serum albumin (< 3 g/dL) were strongly linked to the development of osteoporosis in the lumbar spine, femoral neck, and hip (22). Takako et al. found that 36.4% of patients with osteoporotic vertebral compression fractures (OVCFs) develop malnutrition, an important factor in the reduced activities of daily living and postoperative falls (23). The GNRI is a simple tool for determining a patient’s nutritional status based on height (cm), weight (kg), ideal weight (kg), serum albumin, and other factors (4). BMI is calculated from height and weight. BMI and osteoporosis occurrence are closely related, but not in a simple linear relationship. High BMI (> 26-28 kg/m^2^) as well as low BMI (< 22-24 kg/m^2^) can increase the occurrence of osteoporosis (24–26). In people over the age of 50, a BMI of 23.0-24.9 kg/m^2^ can reduce the risk of osteoporosis and type 2 diabetes (27). Wang et al. (5). also demonstrated that GNRI was positively associated with the lumbar spine, hip, and femoral neck BMD and negatively associated with osteoporosis development in patients with type 2 diabetes. Furthermore, GNRI is a more accurate clinical predictor of osteoporosis occurrence than BMI, albumin, and age. In addition, GNRI was positively correlated with BMD-T scores, body composition, and grip strength in hemodialysis patients, and a high GNRI tended to imply better bone quality (28). Qing et al. reported the relationship between GNRI and BMD in the Chinese elderly through a large cross-sectional study of 1130 participants for the first time. Authors found that higher GNRI values were associated with higher levels of hip BMD but not significantly with lumbar spine BMD (8) Contrarily, we found a significant linear relationship (*P* < 0.001) between either femoral neck BMD or lumbar spine BMD and GNRI based on a larger cross-sectional survey population (1621 with osteoporosis and 6906 in the control population), with higher GNRI being associated with higher femoral neck or lumbar spine BMD. These findings imply that nutritional status affects bone quality throughout the body in older adults.

GNRI was identified as a risk factor for the development of osteoporosis in the elderly by multifactorial logistic regression scores. In the present study, osteoporosis (24.4% vs. 18.9%) was also significantly higher in the elderly with low GNRI than in the high GNRI population. After adjusting for covariates such as age, gender, and diabetes, the findings remained consistent. We investigated the dose-response relationship between GNRI and the risk of osteoporosis and found that GNRI had a non-linear negative correlation with the risk of osteoporosis in the elderly population rather than a simple linear relationship. In short, the pathogenesis of osteoporosis is replicated, and multiple factors are involved in the development of osteoporosis.

There are some limitations associated with this study. First, in this cross-sectional study, it is difficult to speculate on the causal relationship between GNRI and BMD/osteoporosis. Because the NHANES study collected data at a single time point, nutritional data such as serum albumin, height, and weight were only recorded once for all participants, and BMD-T values of lumbar spine and femoral neck were only measured once, potentially resulting in some bias in GNRI and BMD-T scores. Therefore, in the future, we need to conduct multicenter longitudinal clinical trials to confirm our findings, dynamically assess changes in each of the factors that may affect BMD/osteoporosis and lead long-term follow-up to investigate how nutrition levels specifically affect the development and progression of osteoporosis in older adults.

## Conclusion

This study found a significant association between low levels of GNRI and the development of osteoporosis in older adults through a nationwide cross-sectional study. Our analysis of a nationally representative sample suggests that low levels of GNRI are a risk factor for the development of osteoporosis in older adults and can also be used as a predictor of osteoporosis risk in older adults.

## Data availability statement

Publicly available datasets were analyzed in this study. This data can be found here: https://www.cdc.gov/nchs/nhanes/.

## Author contributions

WH carried out the acquisition and interpretation of data and was the major contributor to drafting the manuscript. YQX carried out the clinical data collection and analysis. HWW provided guidance for the revision of the introduction and discussion of the article.KXL guided the statistical method of the article and assisted in drawing revised **Tables 2** and **3**. YQX and WH contributed to the ideas of the article and reviewed the manuscript. All authors contributed to the article and approved the submitted version.

## Conflict of interest

The authors declare that the research was conducted in the absence of any commercial or financial relationships that could be construed as a potential conflict of interest.

## Publisher’s note

All claims expressed in this article are solely those of the authors and do not necessarily represent those of their affiliated organizations, or those of the publisher, the editors and the reviewers. Any product that may be evaluated in this article, or claim that may be made by its manufacturer, is not guaranteed or endorsed by the publisher.
